# Caffeine Controls Glutamatergic Synaptic Transmission and Pyramidal Neuron Excitability in Human Neocortex

**DOI:** 10.3389/fphar.2017.00899

**Published:** 2018-01-04

**Authors:** Amber Kerkhofs, Ana C. Xavier, Beatriz S. da Silva, Paula M. Canas, Sander Idema, Johannes C. Baayen, Samira G. Ferreira, Rodrigo A. Cunha, Huibert D. Mansvelder

**Affiliations:** ^1^Department of Integrative Neurophysiology, Center for Neurogenomics and Cognitive Research, Neuroscience Amsterdam, Vrije Universiteit Amsterdam, Amsterdam, Netherlands; ^2^Center for Neuroscience and Cell Biology, University of Coimbra, Coimbra, Portugal; ^3^Portuguese National Institute of Legal Medicine and Forensic Sciences, Coimbra, Portugal; ^4^Department of Neurosurgery, Neuroscience Amsterdam, VU University Medical Center Amsterdam, Amsterdam, Netherlands; ^5^Faculty of Medicine, University of Coimbra, Coimbra, Portugal

**Keywords:** caffeine, human neocortex, synapses, adenosine, A_1_R, pyramidal neuron

## Abstract

Caffeine is the most widely used psychoactive drug, bolstering attention and normalizing mood and cognition, all functions involving cerebral cortical circuits. Whereas studies in rodents showed that caffeine acts through the antagonism of inhibitory A_1_ adenosine receptors (A_1_R), neither the role of A_1_R nor the impact of caffeine on human cortical neurons is known. We here provide the first characterization of the impact of realistic concentrations of caffeine experienced by moderate coffee drinkers (50 μM) on excitability of pyramidal neurons and excitatory synaptic transmission in the human temporal cortex. Moderate concentrations of caffeine disinhibited several of the inhibitory A_1_R-mediated effects of adenosine, similar to previous observations in the rodent brain. Thus, caffeine restored the adenosine-induced decrease of both intrinsic membrane excitability and excitatory synaptic transmission in the human pyramidal neurons through antagonism of post-synaptic A_1_R. Indeed, the A_1_R-mediated effects of endogenous adenosine were more efficient to inhibit synaptic transmission than neuronal excitability. This was associated with a distinct affinity of caffeine for synaptic *versus* extra-synaptic human cortical A_1_R, probably resulting from a different molecular organization of A_1_R in human cortical synapses. These findings constitute the first neurophysiological description of the impact of caffeine on pyramidal neuron excitability and excitatory synaptic transmission in the human temporal cortex, providing adequate ground for the effects of caffeine on cognition in humans.

## Introduction

Coffee is the second most consumed beverage after water and its main constituent, caffeine, is the most widely consumed drug, improving attention and alertness, and normalizing mood and cognition (Fredholm et al., 1999; Smith, 2002). These central effects of caffeine result from the antagonism of adenosine receptors (Fredholm et al., 1999), in particular A_1_ receptors (A_1_R) and A_2A_ receptors (A_2A_R), which are the main adenosine receptors in the brain (Fredholm et al., 2005). Because of its hydrophobic properties, brain concentrations of caffeine are similar to plasma concentrations (McCall et al., 1982; Fredholm et al., 1999), in the range of 20–70 μM upon moderate intake (Thithapandha et al., 1972; Kaplan et al., 1990; Costenla et al., 2010). These concentrations trigger the maximal psychostimulant effects of caffeine (Bruce et al., 1986). Careful consideration of caffeine concentration is important since most effects of caffeine are bell-shaped, being a psychostimulant with neuroprotective actions at moderate doses and a depressant with deleterious effects at higher doses (Rogers and Dernoncourt, 1998; Smith, 2002; Doepker et al., 2016).

Moderate doses of caffeine antagonize adenosine receptors (Daly et al., 1981; El Yacoubi et al., 2000; Karcz-Kubicha et al., 2003; Huang et al., 2005; Yang et al., 2009; Simões et al., 2016), whereas higher caffeine concentrations act on other targets such as the inhibition of phosphodiesterases, the modification of GABA_A_ receptor function or the release of calcium from intracellular calcium stores (Lopez et al., 1989; McPherson et al., 1991; Fredholm et al., 1999; Boswell-Smith et al., 2006). These are likely associated with the toxic effects of caffeine (Yu et al., 2009). Since A_1_R are inhibitory, reducing excitatory synaptic transmission and neuronal excitability (Dunwiddie and Masino, 2001), whereas A_2A_R are mostly excitatory, facilitating synaptic plasticity processes (Cunha, 2016), it is assumed that the neurostimulant effect of caffeine mostly results from the partial antagonism of A_1_R whereas the neuroprotective effects of caffeine may results from limiting excessive A_2A_R activation (Ferré, 2008; Cunha, 2016). The role of A_1_R in the cerebral cortex has mostly been studied in rodents, where A_1_R are mostly located at synapses (Tetzlaff et al., 1987; Rebola et al., 2003), in particular at excitatory rather than at inhibitory synapses (e.g., Rombo et al., 2015). At excitatory synapses, A_1_R depress synaptic transmission and neuronal excitability through a combined presynaptic action decreasing glutamate release, post-synaptic actions decreasing the activation of glutamate receptors and voltage-sensitive calcium channels (de Mendonça et al., 1995; Klishin et al., 1995) as well as extra-synaptic actions through a decrease of neuronal excitability by controlling potassium rectifier channels (Greene et al., 1985), after-hyperpolarization potentials (Haas and Greene, 1984) and HCN (Li et al., 2011). Both *in vivo* and *in vitro* studies in rodents concluded that the inhibitory effect of A_1_R predominantly results from the presynaptic inhibition of glutamate release (Phillis et al., 1979; Thompson et al., 1992).

Despite the clear effects of caffeine in human subjects on cortical regions resulting in alterations of vigilance, mood, and cognition (Smith, 2002; Doepker et al., 2016), the functional impact of caffeine on neuronal excitability and information flow in the human cerebral cortex is not yet characterized. On a molecular level, moderate doses of caffeine affect A_1_R binding (Boulenger et al., 1982) and occupancy in human cortical neurons (Elmenhorst et al., 2012; Paul et al., 2014). While studies in rodents revealed an ability of caffeine to partially antagonize some A_1_R-mediated effects (Phillis et al., 1979; Qi et al., 2016), responses in the human cortical network might differ as A_1_R have clear inter-species differences, typified by a lower density (Boulenger et al., 1982; Dodd et al., 1986; Fastbom et al., 1987; Svenningsson et al., 1997), a higher affinity for agonists and a lower affinity for antagonists in human *versus* rodent cerebral cortex (Murphy and Snyder, 1982; Ferkany et al., 1986; Maemoto et al., 1997). Therefore, we here delineate the synaptic and subsynaptic localization of A_1_R in the human cerebral cortex, and test how A_1_R affect neuronal excitability and excitatory synaptic transmission. We report how caffeine at realistic concentrations reached in the brain parenchyma and experienced by coffee consumers after 1–2 cups of coffee, affects these neuronal and synaptic A_1_R actions in human neocortex.

## Materials and Methods

### Human Samples

All procedures on human brain resection material that had to be removed for the surgical treatment of deeper brain structures were performed with the approval of the Medical Ethical Committee of the VU University Medical Centre, written informed consent by patients involved, and in accordance with Dutch license procedures and the declaration of Helsinki, as previously described (Verhoog et al., 2016). Human brain samples were also collected at autopsies, performed at the Instituto Nacional de Medicina Legal e Ciências Forenses, which approved all procedures according to the rules of the European Consortium of Nervous Tissues: BrainNet Europe II, to protect the identity of individual donors, as previously described (Pliássova et al., 2016).

### Membrane Preparation and Binding Assays

Total membranes and synaptic membranes (from a synaptosomal preparation) were obtained by isopicnic and gradient centrifugations of homogenized brain tissue, as previously described (Rebola et al., 2005; Pliássova et al., 2016). To determine the enrichment and basic binding characteristics of A_1_R in cortical synapses, we compared saturation binding isotherms of the selective A_1_R antagonist ^3^H-DPCPX (0.1–10 nM; specific activity of 102.1 Ci/mmol; from DuPont NEN) in total and synaptosomal membranes (72–164 μg) incubated for 2 h incubation at room temperature in a buffer containing 50 mM Tris, 1 mM EDTA, 2 mM EGTA, pH 7.4, with adenosine deaminase (4 U/ml, Roche) before filtration through Whatman GF/C filters (Millipore), as previously described (Rebola et al., 2003; Coelho et al., 2006). To estimate the binding affinity of caffeine, we carried out displacement curves of ^3^H-DPCPX binding with caffeine (0.1–300 μM; from Sigma), as previously described (Coelho et al., 2006). Results are expressed as specific binding, determined by subtraction of the non-specific binding, which was measured in the presence of 2 μM 8-{4-[(2-aminoethyl)amino]carbonylmethyloxyphenyl}xanthine (XAC, a mixed A_1_R/A_2A_R antagonist; from Tocris) and normalized per amount of protein (bicinchoninic acid assay). To derive the binding parameters from saturation curves (K_D_ and B_max_ values) the data were fitted by a rectangular hyperbola using the GraphPad Prism software. For displacement binding curves, IC_50_ values were converted to K_i_ values by non-linear fitting of the semi-logarithmic curves derived from the competitions curves.

### Subsynaptic Fractionation and Western Blot Analysis

To separate the extrasynaptic (non-active) zone, presynaptic active zone and post-synaptic fractions from synaptosomes, we used a fractionation method previously described in detail (Rebola et al., 2005; Canas and Cunha, 2016). The efficiency of separation is based on the segregation of different markers in the several fractions: SNAP-25 in the presynaptic active zone, PSD-95 in the post-synaptic density and synaptophysin outside the active zone (extrasynaptic fraction). Western blot analysis was performed with a rabbit anti-A_1_R antibody (1:500, Thermo Scientific), as previously described (Rebola et al., 2003).

### Human Brain Slice Preparation and Electrophysiological Recordings

Human brain slices were derived from resected tissue obtained from patients suffering from mild-to severe forms of epilepsy (*n* = 6, of which 5 were diagnosed with meso-temporal epilepsia, 1 with dysplasia; average age 44.8 ± 10.0). All obtained tissue is derived from the temporal lobe area (*n* = 6), away from the focal area of the epilepsy. Slices were prepared as described previously (Testa-Silva et al., 2014; Verhoog et al., 2016). Briefly, blocks of resected cortical tissue were transported to the laboratory in carbogen-saturated (95% O_2_, 5% CO_2_ at pH 7.4) ice-cold choline-based slicing solution containing (in mM): 110 choline chloride, 11.6 sodium ascorbate, 2.5 KCl, 1.3 NaH_2_PO_4_, 7 MgCl_2_, 0.5 CaCl_2_, 26 NaHCO_3_, 10 glucose. Cortical slices (350–400 μm) were prepared in the same ice-cold solution as used for transport, and then transferred to holding chambers with artificial cerebrospinal fluid (aCSF) containing (in mM) 125 NaCl, 3 KCl, 1.25 NaH_2_PO_4_, 1 MgSO_4_, 2 CaCl_2_, 26 NaHCO_3_, 10 glucose. Here, they were stored for 30 min at 34°C and subsequently at room temperature for at least 1 h before recording.

Following recovery, recordings from cells in deeper layers of the human temporal cortex were made in oxygenated aCSF (flow rate of 2–3 ml/min, 32°C). Whole-cell patch-clamp recordings were made with borosilicate glass pipettes (3–6 MΩ) filled with an intracellular solution containing (in mM): 111 K-gluconate, 8 KCl, 10 HEPES, 4 Mg-ATP, 10 K_2_HPO_4_, 0.4 GTP, 0.2 EGTA. Biocytin (0.5%) was added to all solutions for *post hoc* cell identification, and osmolarity was adjusted to 290–295 mOsm. Pyramidal neurons were visualized with differential interference contrast microscopy, selected based on their large and pyramidal shape and further identified by their spike profile. During recordings, cells were kept at a holding potential close to -70 mV. Recordings were made using MultiClamp 700 A/B amplifiers (Axon Instruments, Sunnyvale, CA, United States), sampling at 10 kHz and low-pass filtering at 3–4 kHz. Recordings were digitized with an Axon Digidata 1440A and acquired using pClamp software (Axon). Acquired data were stored for off-line analysis.

To characterize the electrophysiological properties of pyramidal neurons, we used a step protocol to calculate the input resistance (Rin) as the slope of the linear fit through the current–voltage relationship in 10 pA steps. The membrane time constant was obtained by fitting a single exponential function to the membrane potential deflection in response to a -50 pA current injection. The sag was calculated as the percentage of the difference between transient and stable membrane potentials to a hyperpolarization amplitude of -10 mV after injecting a negative current. The rheobase was defined as the minimal current amplitude of 1 s duration that resulted in the first action potential (AP). Analysis of spike waveforms was performed on single APs elicited by depolarizing threshold current pulses. The AP half-width was defined as the spike width at its half amplitude. To study synaptic transmission, we evaluated spontaneous events that were detected using MiniAnalysis software. The average amplitude and frequency of excitatory post-synaptic potentials (EPSPs) was determined over a time span of 5 min.

The tested drugs, adenosine (20 μM, Sigma), caffeine (50 μM, Sigma) or DPCPX (100 nM, Tocris), were used in concentrations previously tested in rodent preparations (Cunha et al., 1998; Sebastião et al., 2000; Costenla et al., 2010) and were dissolved in aCSF at the desired concentration and bath applied during the experiments.

### Morphological Analysis

After recording, slices were fixed at 4°C for at least 24 h in 100 mM phosphate buffer saline containing 4% paraformaldehyde (pH 7.4), for subsequent neuronal visualization and reconstruction as previously described (Mohan et al., 2015). Slices containing biocytin-filled neuronal pairs were processed using a protocol described previously (Mohan et al., 2015). Slices were incubated in 0.1% Triton X-100 solution containing avidin biotinylated horseradish peroxidase (ABC-Elite; Camon); subsequently, they were reacted using 3,3-diaminobenzidine as a chromogen under visual control until the dendritic and axonal arborization was clearly visible (usually after 2–4 min). Slices were dehydrated and then mounted on slides, embedded in Mowiol and enclosed with a thin coverslip.

Biocytin-labeled neurons were examined under the light microscope. Representative pairs were photographed at low magnification to document the dendritic and axonal arborization. Subsequently, neurons were reconstructed with the aid of Neurolucida software (MicroBright Field).

### Statistical Analysis

Data are means ± SEM. Paired Student’s *t*-test or Wilcoxon signed-rank test (*n* < 10) was used for statistical comparisons between two groups and one-way ANOVA followed by *post hoc* Tukey’s test for statistical comparisons among multiple groups. The normality of the distribution of values was determined by the Kolmogorov–Smirnov test. Statistical significance was set at *p* < 0.05.

## Results

### Synaptic Localization of A_1_R in the Human Cerebral Cortex

**FIGURE 1:**
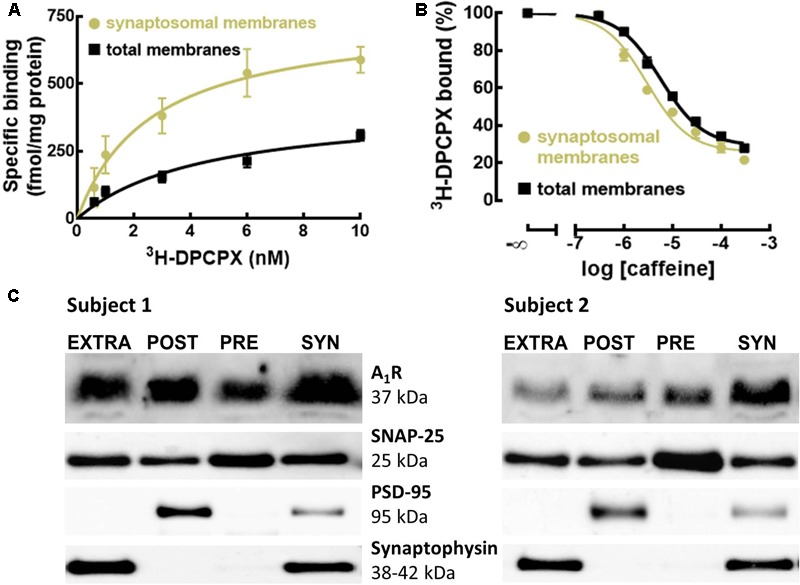
A_1_R are enriched and evenly distributed in human cortical synapses, and have greater affinity for caffeine and selective A_1_R antagonist compared to extra-synaptic A_1_R. **(A)** The selective A_1_R antagonist ^3^H-DPCPX binds with greater affinity and to a larger number of A_1_R in synaptosomal compared to total membranes of the human temporal cortex. Data are mean ± SEM of five subjects. **(B)** Caffeine has a greater affinity to displace the binding of the selective A_1_R antagonist ^3^H-DPCPX (2 nM) from synaptosomal compared to total membranes of the human temporal cortex. Data are mean ± SEM of five subjects. **(C)** Western blots of subsynaptic fractions prepared from synaptosomes (SYN) showing the subsynaptic distribution of A_1_R in the human temporal cortex (*n* = 2). A_1_R are present in all the subsynaptic fractions, outside (EXTRA) and inside the presynaptic active zone (PRE) and post-synaptic density (POST).

To define if A_1_R were enriched in synapses of the human cerebral cortex, as occurs in rodents (Tetzlaff et al., 1987; Rebola et al., 2003), we compared the binding density of the previously validated selective A_1_R antagonist ^3^H-DPCPX (Svenningsson et al., 1997) in membranes from synaptosomes (purified synapses) and in total membranes (mostly representing non-synaptic neuronal and astrocytic membranes) from human neocortical tissue. **Figure 1A** shows that the binding density of A_1_R was larger in synaptosomal membranes (B_max_ = 755 ± 22 fmol/mg protein, *n* = 5) than in total membranes (B_max_ = 421 ± 17 fmol/mg protein, *n* = 5; *p* < 0.001 *versus* synaptosomal membranes; unpaired Student’s *t*-test).

Interestingly, ^3^H-DPCPX displayed a higher affinity to bind A_1_R in synaptosomal membranes (K_D_ = 2.68 nM, 95% confidence interval: 2.12–3.24 nM, *n* = 5) than in total membranes (K_D_ = 4.58 nM, 95% confidence interval: 3.69–5.46 nM, *n* = 5; *p* = 0.0079 *versus* synaptosomal membranes; unpaired Student’s *t*-test). These different binding properties of synaptic *versus* extra-synaptic A_1_R in the human cerebral cortex were confirmed by the different apparent affinity of caffeine for these two populations of A_1_R. In fact, competition curves of ^3^H-DPCPX binding by caffeine (a non-selective adenosine receptor antagonist; Daly et al., 1981) showed that caffeine displaced ^3^H-DPCPX binding with greater efficiency in synaptosomal membranes (K_i_ = 11.53 μM, 95% confidence interval: 7.89–15.17 μM, *n* = 5) than in total membranes (K_i_ = 33.02 μM, 95% confidence interval: 25.18–40.86 μM, *n* = 5; *p* = 0.0079 *versus* synaptosomal membranes; unpaired Student’s *t*-test) (**Figure 1B**). This is suggestive of a possible difference in human cerebral cortical synaptic and extra-synaptic membranes of the homo- or heterodimerization of A_1_R, which has been shown to affect the binding of caffeine and A_1_R antagonists (Ciruela et al., 2006; Gracia et al., 2013).

We further detailed the localization of A_1_R within human cerebral cortical synapses. Using different subsynaptic fractions prepared from synaptosomes of the human temporal cortex, we concluded that A_1_R are present in all the subsynaptic fractions on somatodendritic (post-synaptic) and axon terminal (presynaptic) compartments, inside and outside the presynaptic active zone and post-synaptic density (**Figure 1C** and Supplementary Figure S1). Such a widespread distribution predicts a complex role for the A_1_R in the control of synaptic communication in the human cerebral cortex.

### Adenosine Effects on Intrinsic Membrane Properties of Pyramidal Neurons Are Mediated by A_1_R

**FIGURE 2:**
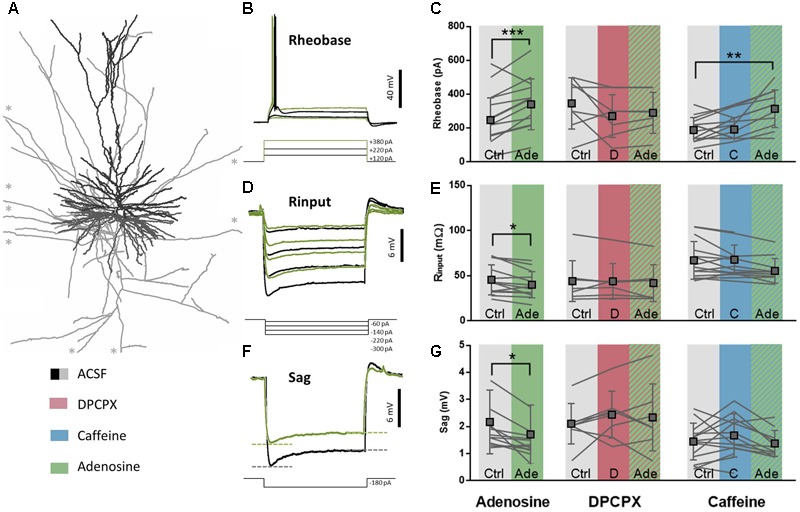
A_1_R-mediated effects on intrinsic membrane properties are moderately affected by caffeine. **(A)** Biocytin reconstruction of deeper layer pyramidal neuron from slice of human temporal cortex showing dendritic (black) and axonal (gray) branches. ^∗^Axonal branches stretch longer than could be depicted here. **(B,E)** Rheobase value of pyramidal neurons increased after adenosine application (20–100 μM, green, **E**, left panel). This effect was blocked by pre-incubation of DPCPX (100 nM, red, **E**, middle panel), but still occurred when caffeine was pre-incubated (50 μM, blue, **E**, right panel). Paired *t*-test comparing adenosine (20–100 μM) to ACSF condition: ^∗∗^*p* < 0.01; ^∗∗∗^*p* < 0.001. Boxes depict average ± SD. **(C,F)** Input resistance of pyramidal neurons decreased after adenosine application (20–100 μM, **F**, left panel). Both DPCPX (100 nM, red, **F**, middle panel) and caffeine (50 μM, **F**, blue, right panel) inhibited adenosine-induced effects (striped green). Paired *t*-test comparing adenosine (20–100 μM) to ACSF condition: ^∗^*p* < 0.05. Boxes depict average ± SD. **(D,G)** The sag of hyperpolarization-activated cationic depolarizing current (I_h_) decreased after adenosine application (20–100 μM, **G**, left panel). Both DPCPX (100 nM, red, **G**, middle panel) and caffeine (50 μM, blue, **G**, right panel) inhibited adenosine-induced effects (striped green). Paired *t*-test comparing adenosine (20–100 μM) to ACSF condition: ^∗^*p* < 0.05. Boxes depict average ± SD.

Adenosine, at non-toxic concentrations (i.e., in low μM range), is known to affect intrinsic membrane properties of layers 2–3 and layer 5 pyramidal neurons in the rodent cerebral cortex through the activation of A_1_R (Phillis et al., 1979; van Aerde et al., 2015). Since A_1_R activation also controls excitability in the human brain (Klaft et al., 2016) by still undefined mechanisms, we first characterized the effect of exogenous adenosine application on membrane properties of pyramidal neurons in the human temporal cortex (**Figure 2A**). On average, adenosine (20–100 μM) did not decrease the resting membrane potential (RMP; control: -67.2 ± 2.3 mV; adenosine: -67.2 ± 3.4 mV; difference: 0.01 ± 3.7 mV, *n* = 12, paired *t*-test: *p* = 0.99), while it did lower the input resistance (R_input_; control: 45.9 ± 16.7 MΩ; adenosine: 40.4 ± 14.5 MΩ; difference: 5.5 ± 6.5 MΩ, *n* = 12, paired *t*-test: *p* = 0.01; **Figures 2C,F**) and increased the current injection needed before firing of the first AP, expressed as the rheobase value (Rheobase; control: 251.7 ± 130.5 pA; adenosine: 344.5 ± 149.9 pA; difference: 92.8 ± 45.0 pA, *n* = 12, paired *t*-test: *p* < 0.0001; **Figures 2B,E**). Altogether these changes lead to a decreased neuronal excitability and a need for a larger input to fire an AP. Furthermore, adenosine decreased the voltage sag (Sag; control: 2.1 ± 1.2 mV; adenosine: 1.6 ± 1.1 mV; difference: 0.45 ± 0.44 mV, *n* = 12, paired *t*-test: *p* = 0.003; **Figures 2D,G**), reflecting an adenosine-induced partial block of HCN channels, as described in rodents (Li et al., 2011). These effects of adenosine were not seen at 5 μM and were concentration-dependent in the range of 20–100 μM.

The effects of adenosine on membrane intrinsic properties were due to A_1_R activation, since the selective A_1_R antagonist DPCPX (100 nM) blocked the effects of adenosine on the rheobase (DPCPX: 275.0 ± 125.5 pA; DPCPX+adenosine: 293.6 ± 120.4 pA; difference: 16.4 ± 25.0 pA, *n* = 7, paired *t*-test: *p* = 0.13; **Figures 2B,E**), input resistance (DPCPX: 42.2 ± 20.5 MΩ; DPCPX+adenosine: 33.0 ± 12.9 MΩ; difference: 2.2 ± 5.5 MΩ, *n* = 7, paired *t-*test: *p* = 0.33; **Figures 2C,F**), and sag (DPCPX: 2.3 ± 1.2 mV; DPCPX+adenosine: 2.5 ± 0.6 mV; difference: 0.1 ± 0.6 mV, *n* = 7, paired *t*-test: *p* = 0.72; **Figures 2D,G**). By itself, DPCPX (100 nM) did not significantly alter these membrane properties: rheobase (control: 350.0 ± 151.2 pA; DPCPX: 275.0 ± 125.5 pA; difference 75.0 ± 133.8 pA, *n* = 8, paired *t*-test: *p* = 0.16; **Figures 2B,E**), Input resistance (control: 44.4 ± 22.7 MΩ; DPCPX: 42.2 ± 20.5 MΩ; difference: 0.2 ± 5.8 MΩ, *n* = 8, paired *t-*test: *p* = 0.92; **Figures 2C,F**) or sag (control: 2.1 ± 0.7 mV; DPCPX: 2.3 ± 1.2 mV; difference: 0.3 ± 0.6 mV, *n* = 8, paired *t*-test: *p* = 0.17; **Figures 2D,G**). This indicates the lack of a constitutive effect of endogenous adenosine on the A_1_R controlling membrane intrinsic properties of pyramidal neurons in human cortical slices.

### Caffeine Does Not Modify Intrinsic Membrane Properties of Pyramidal Neurons

As with DPCPX, there were no effects of caffeine (50 μM) by itself on intrinsic membrane properties of pyramidal neurons in human temporal-cortical slices. The rheobase (control: 192.9 ± 73.4 pA; caffeine: 196.4 ± 69.8 pA; difference 18.2 ± 72.9 pA, *n* = 11, paired *t*-test: *p* = 0.42; **Figures 2B,E**), input resistance (control: 67.5 ± 21.0 MΩ; caffeine: 68.2 ± 16.3 MΩ; difference: 4.1 ± 10.3 MΩ, *n* = 11, paired *t-*test: *p* = 0.21; **Figures 2C,F**) and sag were unaltered (control: 1.4 ± 0.7 mV; caffeine: 1.6 ± 0.8 mV; difference: 0.3 ± 0.6 mV, *n* = 11, paired *t*-test: *p* = 0.12; **Figures 2D,G**). Since the selective A_1_R antagonist DPCPX had no effect by itself and thereby ruled out constitutive activation of A_1_R in human cortical slices, the lack of a direct effect of caffeine on intrinsic membrane properties suggests that A_2A_R are also not constitutively altering intrinsic membrane properties in human pyramidal neurons *in vitro* (reviewed in Cunha, 2016).

We next tested if concentrations of caffeine reached in the brain parenchyma by moderate coffee drinkers (50 μM) could effectively antagonize the A_1_R-mediated control of the intrinsic membrane properties of pyramidal neurons. The effect of exogenously applied adenosine on the rheobase was still observed in the presence of caffeine (50 μM). Thus, when adenosine (20 μM) was added in the presence of caffeine (50 μM), the rheobase increased significantly (control: 192.9 ± 73.4 pA; caffeine+adenosine: 318.2 ± 110.8 pA; difference: 116.4 ± 108.7 pA, *n* = 11, paired *t*-test: *p* = 0.005; **Figures 2B,E**). In contrast, caffeine partially blocked the effects of adenosine on the input resistance (control: 67.5 ± 21.0 MΩ; caffeine+adenosine: 55.8 ± 13.6 MΩ; difference: 4.1 ± 10.3 MΩ, *n* = 11, paired *t-*test: *p* = 0.21; **Figures 2C,F**) and voltage sag (control: 1.4 ± 0.7 mV; caffeine+adenosine: 1.3 ± 0.5 mV; difference: 0.3 ± 0.7 mV, *n* = 11, paired *t*-test: *p* = 0.12; **Figures 2D,G**). This indicates that caffeine concentrations experienced by coffee drinkers can interfere with effects of adenosine on the intrinsic membrane properties of pyramidal neurons in human neocortex.

### Adenosine Controls Synaptic Transmission through A_1_R

**FIGURE 3:**
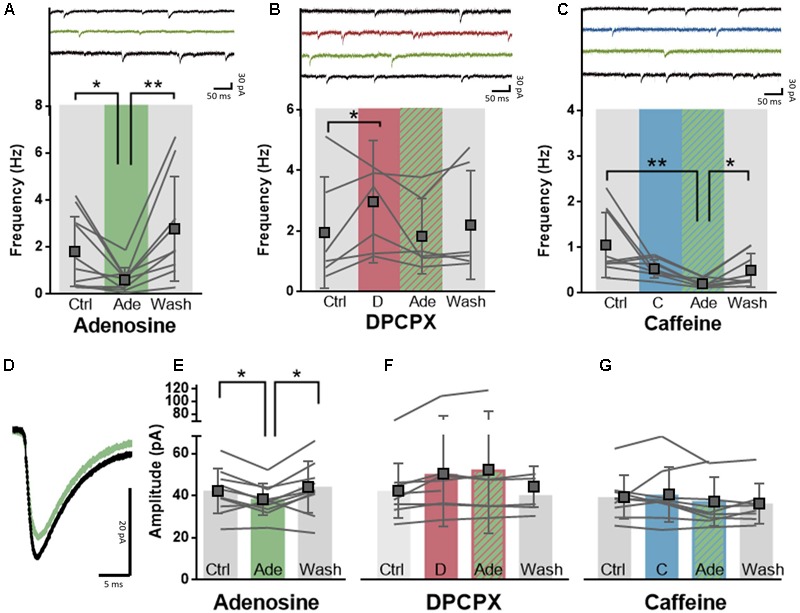
A_1_R-mediated effects of adenosine on synaptic transmission are post-synaptically blocked by caffeine. **(A)** Adenosine (20 μM, green) significantly decreased the frequency of excitatory events onto pyramidal neurons in a reversible manner. Paired *t*-test comparing adenosine (20 μM) to ACSF condition: ^∗^*p* < 0.05; ^∗∗^*p* < 0.01. Boxes depict average ± SD. **(B)** Direct application of DPCPX (100 nM, red) significantly increased the frequency of excitatory events and blocked adenosine-induced inhibition (green). Paired *t*-test comparing DPCPX (100 nM) to ACSF condition: ^∗^*p* < 0.05. Boxes depict average ± SD. **(C)** Caffeine pre-incubation (50 μM, blue) did not affect the potency of adenosine (20 μM, green) to decrease the frequency of excitatory events onto pyramidal neurons. Wash-out of adenosine and caffeine restored the frequency of events. Paired *t*-test comparing adenosine (20 μM) to ACSF condition: ^∗^*p* < 0.05; ^∗∗^*p* < 0.01. Boxes depict average ± SD. **(D)** Example trace of the average effect of adenosine application onto the amplitude of excitatory events onto a pyramidal neuron. **(E–G)** Adenosine (20 μM, green) significantly decreased the amplitude of excitatory events onto pyramidal neurons in a reversible manner **(E)**. Pre-incubation with either DPCPX (100 nM, red, **F**) or caffeine (50 μM, blue, **G**) completely abolished this effect. Paired *t*-test comparing adenosine (20 μM) to ACSF control/wash-out condition: ^∗^*p* < 0.05.

The impact of caffeine on cerebral cortical-mediated behavioral responses may also result from its ability to control glutamatergic synaptic transmission, as observed in rodents (Phillis et al., 1979; Thompson et al., 1992; Costenla et al., 2010; Qi et al., 2016). To evaluate the effect of caffeine on glutamatergic synaptic transmission in the human neocortex, we made whole-cell recordings of large deeper-layer pyramidal neurons of the human temporal cortex and recorded spontaneous excitatory transmission. Adenosine (20 μM) significantly decreased both the amplitude of excitatory events (control: 41.7 ± 10.6 pA; adenosine: 38.1 ± 6.6 pA; difference: 3.9 ± 4.9 pA, *n* = 10, paired *t*-test: *p* = 0.03, **Figures 3D,E**) as well as the frequency of excitatory events (control: 1.8 ± 1.6 Hz; adenosine: 0.6 ± 0.5 Hz; difference: 1.2 ± 1.4 Hz; *n* = 10, paired *t*-test: *p* = 0.02; **Figure 3A**). Both effects were reversible upon washout of adenosine (**Figure 3E**; EPSC amplitude: wash-out: 43.6 ± 12.2 pA; difference vs. adenosine: 5.8 ± 6.6 pA; *n* = 10, paired *t*-test: *p* = 0.02; EPSC frequency: wash-out: 2.8 ± 2.2 Hz; difference vs. adenosine: 2.1 ± 1.8 Hz; *n* = 9, paired *t*-test: *p* = 0.009). The effects of adenosine were mediated by A_1_R, since the selective A_1_R antagonist DPCPX (100 nM) blunted the effect of exogenously added adenosine (20 μM) on frequency (control: 2.0 ± 1.8 Hz; DPCPX+adenosine: 1.9 ± 1.2 Hz; difference: 0.1 ± 0.9 Hz; *n* = 6, paired *t*-test: *p* = 0.77; **Figure 3B**) and amplitude of excitatory post-synaptic events (control: 41.8 ± 12.9 pA; DPCPX+adenosine: 51.7 ± 30.0 pA; difference: 9.7 ± 17.2 pA, *n* = 7, paired *t*-test: *p* = 0.18; **Figure 3F**). DPCPX (100 nM) by itself increased the frequency (control: 2.0 ± 1.8 Hz; DPCPX: 3.0 ± 2.0 Hz; difference: 1.1 ± 0.6 Hz; *n* = 6, paired *t*-test: *p* = 0.01; **Figure 3B**) but not the amplitude of excitatory synaptic events (control: 41.8 ± 12.9 pA; DPCPX: 49.8 ± 24.9 pA; difference: 8.0 ± 13.0 pA, *n* = 8, paired *t*-test: *p* = 0.13; **Figure 3F**), which may suggest a constitutive activity of presynaptic A_1_R inhibiting synaptic release of glutamate.

We next investigated the effect of caffeine concentrations experienced by most coffee drinkers (50 μM) on synaptic transmission in the presence of adenosine. Caffeine (50 μM) blocked the effect of adenosine (20 μM) on the amplitude of spontaneous excitatory events which indicates a post-synaptic effect of caffeine (control: 38.9 ± 10.3 pA; caffeine+adenosine: 36.7 ± 11.5 pA; difference: 2.1 ± 8.9 pA, *n* = 9, paired *t*-test: *p* = 0.49; **Figure 3G**), but did not prevent the effect of adenosine on the frequency of spontaneous events (control: 1.1 ± 0.7 Hz; caffeine+adenosine: 0.2 ± 0.1 Hz; difference: 0.9 ± 0.7 Hz; *n* = 9, paired *t*-test: *p* = 0.007; **Figure 3C**). Similar to the adenosine-only group, effects of adenosine in the case of pre-incubated caffeine could be washed out (wash-out: 0.5 ± 0.4 pA; difference vs. adenosine: 0.3 ± 0.3 pA; *n* = 8, paired *t*-test: *p* = 0.04; **Figure 3C**). In contrast to DPCPX, caffeine (50 μM) by itself had no effect on spontaneous glutamatergic synaptic transmission. It did not modify the amplitude (control: 38.9 ± 10.3 pA; caffeine: 40.1 ± 12.9 pA; difference: 1.1 ± 0.6 pA, *n* = 9, paired *t*-test: *p* = 0.59; **Figure 3G**) or frequency of spontaneous excitatory post-synaptic events (control: 1.1 ± 0.7 Hz; caffeine: 0.6 ± 0.2 Hz; difference: 0.5 ± 0.7 Hz; *n* = 9, paired *t*-test: *p* = 0.06; **Figure 3C**). This may suggest that, in contrast to DPCPX, moderate concentrations of caffeine are not sufficient to interfere with constitutive activity of presynaptic A_1_R. Together, our findings show that caffeine concentrations experienced by coffee drinkers interfere with adenosine signaling when there is an additional adenosine load, allowing to restore excitatory synaptic transmission and pyramidal neuron excitability in the human neocortex.

## Discussion

In this study, we provide the first characterization of the impact of caffeine concentrations experienced by moderate coffee drinkers (50 μM) on neuronal excitability in pyramidal neurons and excitatory synapses of the human temporal cortex. Caffeine had a larger affinity for synaptic A_1_R compared to extra-synaptic A_1_R in the human temporal cortex, which contrasts with the affinity profile of caffeine in the rodent cortex; this difference probably results from a different molecular organization of A_1_R within human cortical synapses compared to rodent cortical synapses. Caffeine interfered with several of the inhibitory A_1_R-mediated effects of adenosine in the human cortex, similar to previous observations in the rodent brain. Caffeine blunted the effects of adenosine on intrinsic membrane properties and prevented the amplitude reduction of excitatory synaptic transmission through antagonistic actions at post-synaptic A_1_R.

A distinct interaction of caffeine with A_1_R in the human cerebral cortex compared with rodent cortex is in agreement with several previous studies describing differences of binding properties of brain A_1_R between humans and different animal models (Boulenger et al., 1982; Murphy and Snyder, 1982; Dodd et al., 1986; Ferkany et al., 1986; Fastbom et al., 1987; Maemoto et al., 1997; Svenningsson et al., 1997). Our results confirmed previous evidence that the binding density of A_1_R in the temporal cortex is lower in humans compared to mice or rats (Boulenger et al., 1982; Dodd et al., 1986; Fastbom et al., 1987; Svenningsson et al., 1997). We also confirmed previous evidence that human A_1_R display a different affinity for xanthine-based selective antagonists, which tend to have lower affinity in humans compared to rodents (Murphy and Snyder, 1982; Ferkany et al., 1986; Maemoto et al., 1997). Despite these different pharmacological properties, human A_1_R seem to exert an overall inhibitory effect in the cerebral cortex that is similar to that observed in rodents: in fact, the exogenous administration of adenosine decreased the excitability of pyramidal neurons in the human temporal cortex in a manner similar to the inhibition previously reported to occur through A_1_R activation in the cerebral cortex of rodents (Phillis et al., 1979; Li et al., 2011). In rodents (Thompson et al., 1992), tonic adenosine inhibition is dominated by a A_1_R-mediated inhibition of excitatory transmission. In human neocortex, both intrinsic membrane properties of pyramidal neurons as well as excitatory synaptic transmission received by these neurons, were inhibited by somatodendritic post-synaptic A_1_R. Whether one of these mechanisms dominates effects of adenosine on cortical overall excitability in human neocortex cannot be concluded based on our present results. We did find a high density of A_1_R in human cortical synapses, both at somatodendritic (post-synaptic) and axon terminals (presynaptic) compartments, as well as a greater affinity for DPCPX of synaptic A_1_R in comparison with extra-synaptic A_1_R, which may point to a stronger control of synaptic transmission by A_1_R. Since one of the few factors that has been documented to regulate the affinity of A_1_R for antagonists is their relative homomerization (Gracia et al., 2013) or heteromerization (Ciruela et al., 2006), the present findings are suggestive of a different molecular arrangement of A_1_R in synapses of the human cerebral cortex.

To characterize the impact of caffeine on human cortical neurons, we used a concentration of caffeine within the range of concentrations reached by caffeine in the brain parenchyma upon moderate consumption of caffeine (20–70 μM) (Thithapandha et al., 1972; Kaplan et al., 1990; Costenla et al., 2010; Duarte et al., 2012; Silva et al., 2013), which are similar to plasma concentrations of caffeine that cause maximal psycho-activating responses in humans (Bruce et al., 1986). Many studies have described neuronal effects of caffeine using high millimolar or submillimolar concentrations of caffeine (Lee et al., 1987; Martin and Buno, 2003; Margineanu and Klitgaard, 2004; Vyleta and Smith, 2008; Grigoryan et al., 2012), which represent toxic effects of caffeine found in extreme cases of caffeinism (Gilliland and Andress, 1981). Using concentrations of caffeine realistic for normal human consumption, we found that caffeine only targets inhibitory A_1_R, since caffeine mimics the effects of DPCPX. This is in agreement with PET imaging studies that documented a decreased occupancy of cortical A_1_R in subjects consuming caffeine (Elmenhorst et al., 2012; Paul et al., 2014). Importantly, we now report that caffeine displays a greater affinity for synaptic A_1_R compared to extra-synaptic A_1_R. This may suggest that synaptic A_1_R are more strongly affected by caffeine and that interference with inhibitory effects of adenosine on transmission at excitatory synapses is a major effect in the human cerebral cortex. In contrast to effects in rodent neocortex, we find that effects of caffeine in human cortical neurons interfered both with adenosine effects on neuronal excitability and excitatory synaptic transmission through post-synaptic A_1_R, while in rodents, caffeine mostly controls synaptic transmission through presynaptic A_1_R and affect the release of excitatory neurotransmitters (Phillis et al., 1979; Greene et al., 1985). Nevertheless, also in rodent neocortex, caffeine can exert a post-synaptic effect (Greene et al., 1985; Simons et al., 2011) in accordance with the ability of post-synaptic A_1_R to induce robust responses in rodent cortical neurons (van Aerde et al., 2015; Qi et al., 2016). Differences in effects of caffeine in human and rodent neocortex may result from the previously discussed different molecular organization of A_1_R or to a different subsynaptic distribution of A_1_R in rodent and human cortical synapses; thus, A_1_R seem to be more abundant presynaptically in rodent synapses (Tetzlaff et al., 1987; Rebola et al., 2003), whereas we find here in human neocortex an even distribution of A_1_R in all subsynaptic fractions.

For our experiments, we used neocortical tissue derived from epileptic patients that underwent surgery for treatment of deeper brain structures. Although the resected tissue was from regions outside the focal area of epilepsy or tumor, and was not part of the disease, our observations could be affected by disease and medication history. Earlier, adenosine A_1_R density was shown to be up- or downregulated in surrounding neocortical areas of patients suffering from temporal lobe epilepsy (Angelatou et al., 1993; Glass et al., 1996) and adenosine A_1_R agonists are explored as therapeutic targets to treat epilepsy (Boison, 2016). Therefore, the possibility that epileptic conditions affect the adenosine neuromodulation system in the cerebral cortex (Rebola et al., 2005) should be considered as a factor influencing the presently reported effects of caffeine. However, the impact of caffeine in epileptic conditions is still largely unclear (Dworetzky et al., 2010; Samsonsen et al., 2013) and seems to be more evident in the developing than in the mature brain (e.g., Tchekalarova et al., 2006), with reports of proconvulsant (e.g., Chu, 1981) and anticonvulsant effects of caffeine (e.g., Rigoulot et al., 2003). It is also important to keep in mind that the present study focused on the human temporal cortex and it remains to be confirmed if the effects of caffeine are similar in other cortical regions, although both the density of A_1_R and the occupancy of A_1_R by caffeine seem to be similar in different cortical areas (Svenningsson et al., 1997; Diukova et al., 2012; Elmenhorst et al., 2012; Park et al., 2014).

The present characterization of the effects of a physiological concentration of caffeine (50 μM) on the human temporal cortex provides an important insight in the subtle effects of cortical exposure to human caffeine consumption. By acting mainly post-synaptically, caffeine is able to counteract adenosine-induced inhibition of the received excitatory signal. This provides a mechanism to explain the enhancement by caffeine on human cognition (Borota et al., 2014), which is not readily observed in adult rodents (e.g., Dall’Igna et al., 2007; Duarte et al., 2012; Espinosa et al., 2013; Kaster et al., 2015; reviewed in Cunha and Agostinho, 2010), where caffeine intake mostly normalizes rather than bolsters learning and memory performance. Thus, these findings provide the first neurophysiological description of the impact of caffeine on excitatory synaptic transmission in the human temporal cortex, providing adequate ground for the effects of caffeine in normal consumption amounts on cognition in humans.

## Author Contributions

AK, HM, RC, and SF designed the research; AK, AX, BdS, and PC performed the experiments; RC performed the research; JCB and SI provided the resources; AK, HM, and RC wrote the manuscript. All authors commented on the manuscript.

## Conflict of Interest Statement

RC is a scientific consultant for the Institute for Scientific Information on Coffee. The other authors declare that the research was conducted in the absence of any commercial or financial relationships that could be construed as a potential conflict of interest.
