# Supplementary figures and images for: Caffeine Controls Glutamatergic Synaptic Transmission and Pyramidal Neuron Excitability in Human Neocortex
Source: Front Pharmacol. 2018 Jan 4;8:899. doi: 10.3389/fphar.2017.00899 (PMC5758559; doi:10.3389/fphar.2017.00899)

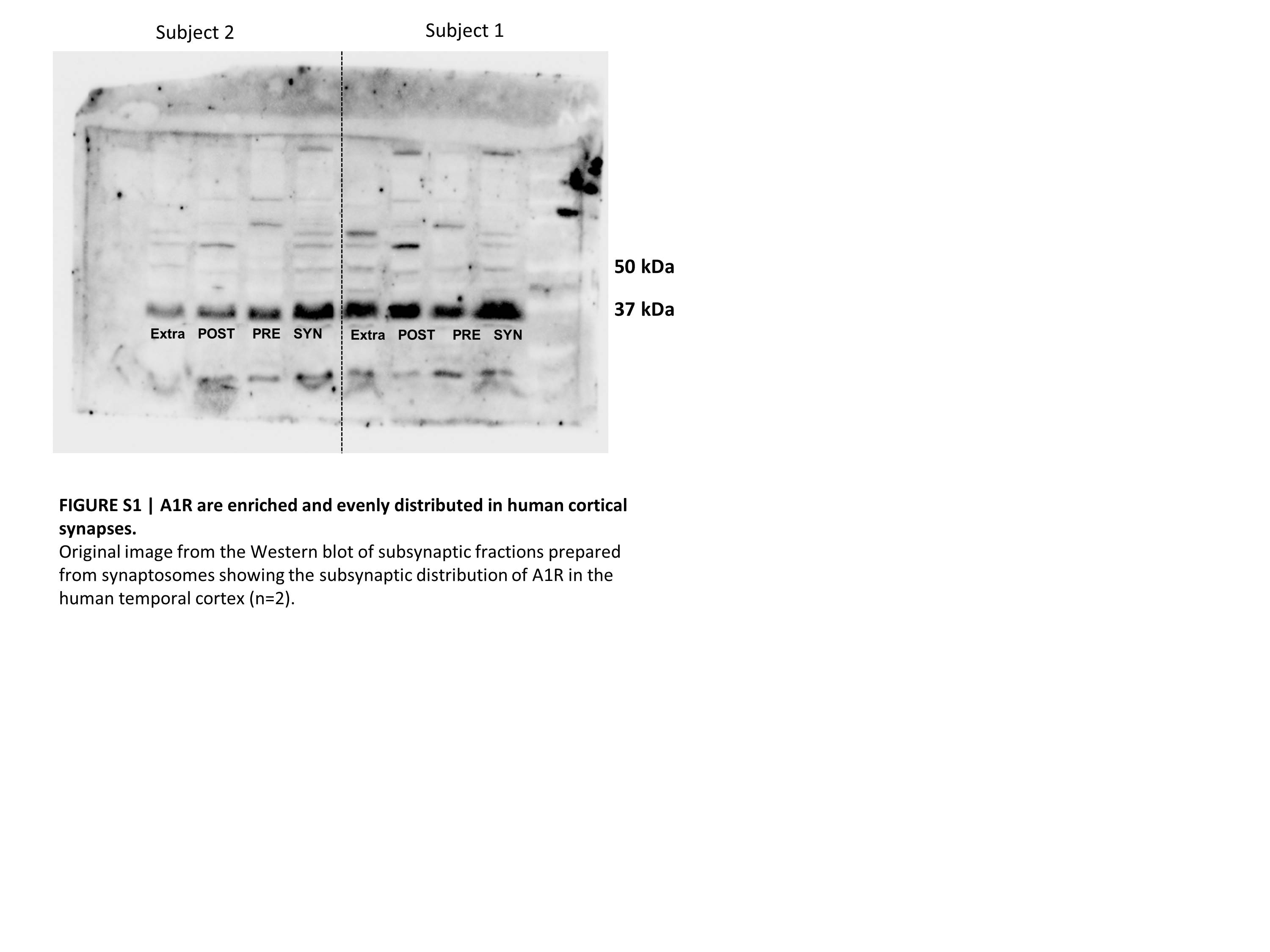

Supplement: Supplementary file 1 [file Image_1.jpg]
